# Characteristics of Orthodontic Treatment in Cancer Survivors: A Systematic Review

**DOI:** 10.3390/jcm13102858

**Published:** 2024-05-12

**Authors:** Nikolaos Karvelas, Ioannis Ntanasis-Stathopoulos, Miltiadis A. Makrygiannakis, Maria Gavriatopoulou, Eleftherios G. Kaklamanos

**Affiliations:** 1 Faculty of Dental Medicine, University of Medicine and Pharmacy “Grigore T. Popa”, 700115 Iasi, Romania; karvelas93@gmail.com; 2 School of Medicine, National and Kapodistrian University of Athens, 11527 Athens, Greecemgavria@med.uoa.gr (M.G.); 3 School of Dentistry, National and Kapodistrian University of Athens, 11527 Athens, Greece; 4 School of Dentistry, European University Cyprus, 2404 Nicosia, Cyprus; kaklamanos@yahoo.com; 5 School of Dentistry, Aristotle University of Thessaloniki, 54124 Thessaloniki, Greece; 6 Hamdan Bin Mohammed College of Dental Medicine (HBMCDM), Mohammed Bin Rashid University of Medicine and Health Sciences (MBRU), Dubai P.O. Box 505055, United Arab Emirates

**Keywords:** orthodontics, cancer, cancer survivors

## Abstract

**Background:** Survival rates of cancer patients have increased globally and across age groups. Challenges arising from craniofacial growth-development disturbances and dental abnormalities might warrant modifications to standard orthodontic pathways of care. **Objective:** The aim of this study was to systematically summarize and critically assess the available literature regarding the characteristics of orthodontic treatment in cancer survivors. **Materials and Methods:** A systematic search was conducted in seven databases for studies on malignant tumor survivors having undergone orthodontic intervention with fixed appliances following cancer treatment up to August 2023. The outcomes of interest included quantitative data regarding various characteristics of orthodontic treatment and the post-treatment period. The risk of bias was assessed individually with the Newcastle-Ottawa scale. **Results:** Out of 347 records, 4 cohort studies were eventually included in the qualitative synthesis. Leukemia was the most common malignancy type, with treatment involving mainly chemotherapy and/or radiotherapy. The duration of orthodontic treatment in cancer survivors varied. Occlusal results, quality of life, and satisfaction were comparable to healthy peers. However, in some survivors’ groups, treatment was shorter and the final results were compromised. Root resorption and oral mucositis were reported among the treated cancer survivors. Reduced occlusal outcome stability during the retention period was also reported. **Conclusions:** Overall, the duration of orthodontic treatment varied among cancer survivors. The occlusal results achieved were similar to those of their healthy peers, though potentially less stable. Patient-reported outcomes did not differ significantly between cancer survivors and healthy individuals treated orthodontically.

## 1. Introduction

Cancer in young populations is rare but still constitutes a major health problem [1,2]. Over the last 30 years, the international burden of cancer in children of up to 14 years of age has been declining, while opposite trends have been observed in adolescents and young adults aged up to 39 years [3,4]. Fortunately, death rates have decreased globally and across age groups [3,4]. In Europe, it has been estimated that almost eighty percent of affected young individuals survive at least five years, with the survival duration in certain cases being even longer [5,6] and continuously improving thanks to new treatment and support regimens [7]. In 2020, the European childhood, adolescent, and young adult cancer survivors’ population was estimated to be 500,000 individuals, and this number is expected to rise by 12,000 each year [8,9].

After overcoming their initial condition, young cancer survivors may experience medical complications and psychosocial repercussions [10,11,12]. As a result, new projects have emerged to support survivors’ medical and quality of life issues [7,13,14]. Moreover, in addition to the effects on surviving children and adolescents’ general growth and development [15,16], chemo- and radiotherapy for cancer might also affect patients’ oral and dental health. Cancer survivors present varying types of malocclusions, i.e., problems with the alignment of teeth and jaws, as well as increased treatment needs warranting orthodontic treatment, i.e., specialist intervention to move teeth and align the jaws. Furthermore, issues and challenges arising from craniofacial growth and development disturbances, as well as the ensuing dental abnormalities and the effects of chemo- and radiotherapy on oral and perioral anatomical structures, might warrant modifications to standard orthodontic pathways of care [17,18,19,20,21,22,23,24,25]. Thus, specialized knowledge and information is needed [26].

### Objective

The aim of this study was to systematically summarize and critically assess the available literature regarding the characteristics of orthodontic treatment in cancer survivors.

## 2. Materials and Methods

### 2.1. Protocol and Registration

This review was based on a protocol that was developed, conducted, and reported according to relevant methodological guidelines [27,28,29,30,31]. The protocol was registered retrospectively in the Open Science Framework (https://osf.io/z7mg5/) (created on 21 August 2023, accessed on 24 March 2024). As this study is a systematic review, ethical approval was not required.

### 2.2. Eligibility Criteria

The eligibility criteria for the present review followed the Population, Intervention, Comparison, Outcome, and Study Design (PICOS) domains (Table 1).

We searched for studies on malignant tumor survivors having undergone orthodontic intervention with fixed appliances following cancer treatment. The outcomes of interest included quantitative data regarding various characteristics of orthodontic treatment and the post-treatment period (e.g., treatment duration, treatment success or compromise, data on occlusal indices, unwanted sequelae and complications, relapse, as well as patient-reported outcomes like quality of life, satisfaction with treatment, etc.). The potentially eligible articles could include comparisons between cancer groups with alternative interventions with control groups of healthy orthodontic patients or could involve no control groups. Case reports, editorial letters, systematic reviews, and animal studies were excluded.

### 2.3. Information Sources and Search Strategy

A systematic search was conducted by NK in 7 databases (Medline (PubMed), CENTRAL (Cochrane Library; includes records from Embase, CINAHL, ClinicalTrials.gov, WHO’s ICTRP, KoreaMed, Cochrane Review Groups’ Specialized Registers, and records identified by hand-searching), Cochrane Database of Systematic Reviews (Cochrane Library), Scopus, Web of Knowledge (including Web of Science Core Collection, KCI Korean Journal Database, Russian Science Citation Index, SciELO Citation Index, and Zoological Record), ClinicalTrials.gov (U.S. National Library of Medicine), and ProQuest Dissertation and Theses (ProQuest)) in order to locate potentially eligible papers up to 11 August 2023. The search strategy was based on PubMed and was adapted for the other databases (Table 2).

There was no limitation regarding language or year of publication. An additional search was performed in the reference lists of the eligible articles (NK and MAM). After the removal of the duplicates using EndNote’s duplicate identification strategy (EndNote X9, Clarivate, Philadelphia, PA, USA), they were then removed manually by EGK, NK, and MAM, who screened the retrieved studies and applied the inclusion criteria independently. In case of disagreement, another author (EGK) made an evaluation so as to settle on a consensus.

### 2.4. Study Selection, Data Collection, and Data Items

The located records were checked independently by NK and MAM. In any case of an unclear abstract, the full text was assessed. The following information was extracted from the finally eligible studies in predetermined forms: bibliographic details of the study; details on study design and verification of study eligibility; cancer survivors’ characteristics where available (number, age, gender, type of cancer, cancer treatment, the time elapsed since treatment); characteristics of treatment and the post-treatment period, to include orthodontic diagnosis, time elapsed since cancer treatment, treatment mechanics, possible extractions carried out, the use of additional interventions (e.g., hyrax), treatment duration, treatment success/compromised treatments/early conclusion, data on occlusal outcomes/indices, unwanted sequelae, and complications, relapse, quality of life, satisfaction, etc. If clarifications were needed regarding the published data, or additional material was required, then attempts to contact the corresponding authors through emails were made.

### 2.5. Risk of Bias Assessment

The risk of bias was assessed by NK and MAM using the Newcastle-Ottawa Scale in all the above-mentioned processes. Disagreements were settled by discussion with EGK; following the relevant suggestions, kappa statistics were not calculated [32].

### 2.6. Data Synthesis, Risk of Bias across Studies, and Additional Analyses

Data synthesis, risk of bias across studies analyses, and additional analyses for “small-study effects” and publication bias were not performed finally due to the lack of an adequate number of studies and the variability of the available information [31].

## 3. Results

### 3.1. Study Selection

Following the previously described search procedures, a total of 347 records were identified. After the removal of 97 duplicate records, we excluded a further 242 on the basis of their title and/or abstracts. Subsequently, eight papers were investigated for eligibility and four further records were excluded (Table 3).

**Figure 1:**
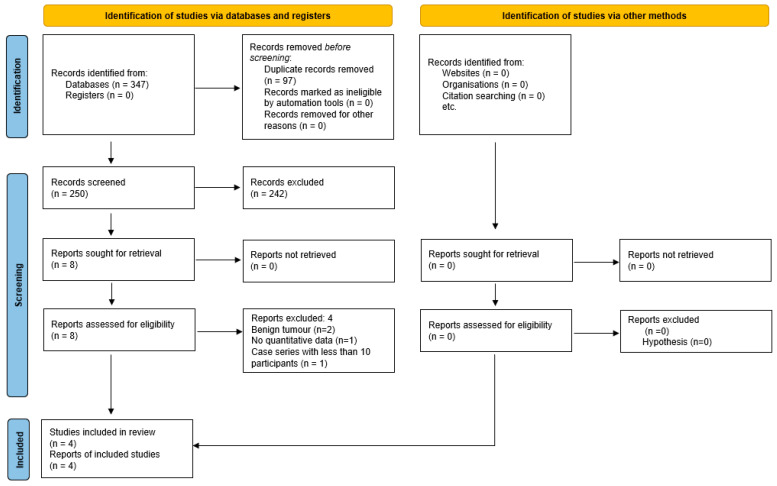
Prisma diagram of the included studies.

Eventually, four articles met all the inclusion criteria and were included in this systematic review (Figure 1) [33,34,35,36].

### 3.2. Cancer Survivors and Orthodontic Treatment Characteristics

The characteristics of the included studies are presented in Table 4.

One case-series study [33] and three cohort studies were located [34,35,36]. Leukemia was the most common malignancy type among cancer survivors, with treatment involving mainly chemotherapy (usually with cyclophosphamide or methotrexate) and/or radiotherapy. Orthodontic treatment involved a variety of skeletal classes and orthodontic problems, and used fixed appliances, while some cases involved extractions or adjuncts such as hyrax. The reported periods of time elapsed between cancer treatment and orthodontic intervention included median or mean values of 8–9 years.

Most treated groups of cancer survivors concluded treatment in approximately a year, while Mitus-Kenig et al. [35] included a cancer survivor—treated group with a mean treatment duration of 19.3 months, and Mitus-Kenig et al. [36] reported on patients treated for mean of 17.6 months. In some patients, the treatment had to be concluded early due to the relapse of the malignancy [33]. Compromised results were reported in the included studies [33,35]; however, the final weighted-PAR Index and the reduction in the weighted-PAR Index were not observed to differ between a cancer survivor group and a control group CG matched for age, gender, and malocclusion [35]. The same was noted by Mitus-Kenig et al. [36] for the final weighted PAR and ICON indices when compared between treated cancer survivors and a matched group of healthy patients [36]. Oral mucosa inflammation [33,34,35] and root resorption [34,35] were reported among the treated cancer survivors. No root resorption was encountered in a cancer survivor group completing treatment within 12 months [35].

Cancer survivors demonstrated significantly more post-orthodontic treatment changes following a period of three years in retention, compared to a controlled group matched for age, gender, malocclusion, and treatment duration. Initial weighed-PAR and ICON indices did not differ between the compared groups. According to the ICON index, 50% of patients in both groups were graded as being of moderate complexity, while 36.5% in cancer survivors and 38.5% in the control group involved cases graded as being either difficult or very difficult. Immediately after orthodontic treatment, the weighed-PAR and ICON indices did not differ either. However, after a period of 3 years in retention, the cancer survivors group demonstrated statistically significant increases in the above-mentioned indices [36].

### 3.3. Patient-Reported Outcomes

Three of the located papers elaborated on patient-reported outcomes from cancer survivors subjected to orthodontic treatment.

The oral health-related quality of life (OHRQoL), as assessed with the 14-item Oral Health Impact Profile (OHIP-14) total and impact scores, did not differ between cancer survivors and healthy control patients before treatment, 2 weeks and 3 months into treatment, or following the removal of appliances [34]. Only psychological discomfort measured during treatment was significantly higher in cancer survivors. Moreover, in the latter group, males reported a significantly lower OHRQoL during the treatment compared to females, a finding not observed in the control group. A similar finding by Mitus-Kenig et al. [36] was that the assessed patient satisfaction was at a similar level before treatment, at the end of treatment, and following 3 years in retention, despite more post-treatment changes observed in the survivors’ group.

Mitus-Kenig et al. [35] compared two groups of cancer survivors; one was treated in a standard manner, while the other was classified as a rapid treatment group. The latter included patients with a risk of hospitalization or oncological examinations potentially influencing orthodontic treatment within a year, who as such were scheduled to finish orthodontic intervention in 12 months. The OHRQoL (OHIP-14 total and impacts scores) did not differ between these groups before treatment, 2 weeks and 3 months into treatment, or following the removal of appliances.

### 3.4. Risk of Bias within Studies

The risk of bias assessments with the Newcastle-Ottawa Scale are presented in Table 5.

All studies were allocated stars for the investigated domains, except for Mitus-Kenig et al. [33,35], which could not receive ratings for the “Selection of the non-exposed cohort” and the “Comparability of cohort on basis of design or analysis” domains, since they did not involve comparisons to healthy, “non-exposed” control groups.

## 4. Discussion

Despite the low frequency of cancer among young populations, it still remains a significant health concern [1,2]. In Europe, it has been estimated that nearly 80% of young individuals diagnosed with cancer survive for a minimum of five years, and in some instances, survival may extend even further [5,6]. This prolonged survival is attributed to the ongoing advances in treatment and supportive care programs [7]. Therefore, it is to be expected that some patients who have survived cancer will seek care in orthodontic offices. To the best of our knowledge, this is the first systematic review to investigate orthodontic treatment characteristics in cancer survivors.

The retrieved material revealed that the duration of orthodontic treatment in cancer survivors varies. Patient groups with a mean treatment duration between 18 and 20 months were reported with final occlusal results similar to those attained in healthy individuals, while approximating to the mean duration of the orthodontic treatment reported for healthy patients [37]. Certain survivor cohorts received treatment for shorter periods, and in some cases the final results were compromised, either because treatment objectives were set as such from the beginning of treatment, as an attempt to mitigate the exacerbation of adverse effects, or as a result of the premature termination of treatment due to a recurrence of the malignancy. Root resorption was reported among the treated cancer survivors but not in the group completing treatment within 12 months [35]. A possible explanation could be that a lengthier orthodontic treatment can lead to increased root resorption [38]. Oral mucositis—a common finding in cancer survivors—was also a consistent finding among the included studies. Photobiomodulation has been reported to be effective in the treatment of oral mucositis [39].

Although no specific correlation has been observed between the type of malignancy and orthodontic diagnosis, several effects of chemo- and radiotherapy on oral and perioral anatomical structures have been observed [24,25]. Impaired root growth and microdontia are the most commonly reported defects [24]. Chemotherapy has also been associated with an increased risk for tooth agenesis compared to healthy controls. Other unwanted sequelae include tooth discoloration, arrested tooth development, enamel hypoplasia, premature apexification, and decreased salivary flow rate. Moreover, worse oral hygiene and increased caries experience compared to controls was noted [25]. Skeletal growth may also be influenced by anti-cancer therapy. Age, dose exposure, and location seem to be significant factors. The mandible has been reported to be four times more sensitive than the maxilla [40]. It has also been observed that antineoplastic treatment may affect occlusion development; increased occurrence of posterior cross-bites has been reported [20]. There is also evidence that oncological treatment has a causal relationship with several other inadvertent effects, such as mucositis, affecting every location of the oral mucosa; infections; neurological disturbances and dysgeusia; hyposialia and xerostomia; bleeding tendencies; and osteonecrosis development [41]. Longo et al. concluded that antineoplastic therapy in patients with childhood cancer has a negative impact on the periodontium and various microbiological parameters [42]. Fifty-three percent of patients who underwent total body irradiation at a young age had a decreased mouth opening and reduced width of translational movements. Also, in 84% of such patients, symptoms of craniomandibular dysfunction were detected [43]. The risk of such adverse effects increases in younger children and with radiation dosages more than 4 Gy [40,44,45]. Dental agenesis and microdontia have been correlated with the patient age at the time of radiotherapy, dose, and length of irradiation, as well as the tumor site [40].

Mitus-Kening et al. [36] reported a decline in outcome for stability in a 3-year retention period of cancer survivors, which indicates the need for a more extensive follow-up to reduce the risk of post-treatment changes [36]. These changes could be attributed to a decreased resistance in tooth movement due to the reduced density of bone following cancer treatment [46]. It could also be the result of the impact of chemotherapeutic drugs per se on the remodeling of the alveolar bone, which in turn could affect the incidence of post-orthodontic treatment changes. Recent systematic reviews have reported on medications that may affect the rate of tooth movement and post-orthodontic treatment changes [47,48,49]. Nevertheless, currently, no evidence exists regarding the direct impact of chemotherapy-related drugs or radiotherapy on the rate of tooth movement, or relapse.

An important finding of the present review was that although complications might arise because of orthodontic treatment, the quality of life of survivors is similar to that of their healthy peers. The oral health-related quality of life (OHRQoL) was not reported to differ between cancer survivors and healthy control patients before treatment, 2 weeks and 3 months into treatment, or following the removal of appliances [34]. Only psychological discomfort measured during treatment was significantly higher in cancer survivors. The findings regarding patient satisfaction up to 3 years in retention were similar, despite more post-treatment changes observed in the survivors’ group [36]. These findings for the survivors having undergone orthodontic treatment are encouraging, since cancer survivors in Europe have been reported to have a lower QoL than their peers [50].

Despite being outside the direct scope of this systematic review, efforts were made by the authors to summarize some points derived from the located information that could be relevant to an orthodontist treating cancer survivors.

- Orthodontic intervention is well tolerated and should preferably start after consulting with the treating physician, since there is a risk of relapse and cancer recurrence.
- Reasonable treatment compromises and simple mechanics could be considered in specific cases, keeping the treatment duration limited, and potentially decreasing the risk of root resorption.
- The use of appliances that do not interfere with radiologic investigation like MRIs should be considered in coordination with the treating physician.
- Special attention should be given to the prevention and management of oral mucositis. Close follow-up during the retention period would be advisable.

## 5. Strengths and Limitations

Despite adhering to the most current methodological guidelines, which constitutes an important strength, this systematic review presents limitations, mainly stemming from the nature of the included studies. Specifically, the limited number of studies, the different oncological diagnoses and treatments of the patients, along with the different orthodontic diagnoses and management, as well as the variability among the outcomes, all significantly increase the heterogeneity among the included studies. All the included studies were retrospective; thus, their results are potentially subject to a bias inherent to the retrospective design. In addition, all these studies were conducted by the same author group in the same country, a fact that may jeopardize the generalizability of the outcomes. Moreover, patient samples may overlap; despite efforts, we were unsuccessful in contacting the author group to gain further details. Therefore, the outcomes should be evaluated cautiously.

## 6. Conclusions

Overall, the duration of orthodontic treatment varied among cancer survivors. The occlusal results achieved were similar to those of their healthy peers, though potentially less stable. The OHRQoL did not differ significantly between cancer survivors and healthy individuals treated orthodontically. The literature on this topic is limited and there is a need for larger prospective cohort studies and clinical trials in the field.

## Figures and Tables

**Table 1:** Eligibility criteria.

Domain	Inclusion Criteria	Exclusion Criteria
**Participants**	▪ Malignant tumor survivors having undergone orthodontic intervention following cancer treatment.	▪ Benign tumor survivors
**Interventions**	▪ Any kind of orthodontic intervention with fixed appliances.	▪ No orthodontic intervention with fixed appliances.
**Comparisons**	▪ No control groups. ▪ Control group of healthy orthodontic patients. ▪ Comparisons between cancer groups with alternative interventions.	
**Outcomes**	▪ Quantitative data regarding characteristics of treatment and post-treatment period (e.g., treatment duration, treatment success/compromised treatments/early conclusion, data on occlusal indices, unwanted sequelae, and complications, relapse, quality of life, satisfaction, etc.).	▪ No quantitative data regarding characteristics of treatment and post-treatment period
**Study design**	▪ Randomized clinical trials, cohort and case-control studies, case series.	▪ Animal studies. ▪ Case reports, editorial letters, studies with fewer than 10 participants (Hulley et al., 2013). ▪ Systematic reviews and meta-analyses.

Hulley SB, Cummings SR, Browner WS, Grady DG, Newman TB (eds). Designing Clinical Research 4th ed. Lippincott Williams & Wilkins, 2013.

**Table 2:** Databases searched (up to 11 August 2023), strategies used, and hits per database.

Database	Search Strategy	Hits
**Medline via PubMed**	(chemotherapy OR chemo OR ctx OR radiotherapy OR radiation OR irradiation OR “x-ray therapy” OR actinotherapy) AND (cancer OR oncol* OR surviv* OR leukemia OR neoplasm* OR tumor OR tumour OR malign*) AND orthodontic*[ti]	**85**
**Cochrane Central Register of Controlled Trials**	(chemotherapy OR chemo OR ctx OR radiotherapy OR radiation OR irradiation OR “x-ray therapy” OR actinotherapy) AND (cancer OR oncol* OR surviv* OR leukemia OR neoplasm* OR tumor OR tumour OR malign*) AND orthodontic* in Record Title OR (chemotherapy OR chemo OR ctx OR radiotherapy OR radiation OR irradiation OR “x-ray therapy” OR actinotherapy) AND (cancer OR oncol* OR surviv* OR leukemia OR neoplasm* OR tumor OR tumour OR malign*) AND orthodontic* in Abstract—(Word variations have been searched)	**17**
**Cochrane Database of Systematic Reviews**	(chemotherapy OR chemo OR ctx OR radiotherapy OR radiation OR irradiation OR “x-ray therapy” OR actinotherapy) AND (cancer OR oncol* OR surviv* OR leukemia OR neoplasm* OR tumor OR tumour OR malign*) AND orthodontic* in Record Title OR (chemotherapy OR chemo OR ctx OR radiotherapy OR radiation OR irradiation OR “x-ray therapy” OR actinotherapy) AND (cancer OR oncol* OR surviv* OR leukemia OR neoplasm* OR tumor OR tumour OR malign*) AND orthodontic* in Abstract—(Word variations have been searched)	**1**
**Scopus**	TITLE-ABS ((chemotherapy OR chemo OR ctx OR radiotherapy OR radiation OR irradiation OR “x-ray therapy” OR actinotherapy) AND (cancer OR oncol* OR surviv* OR leukemia OR neoplasm* OR tumor OR tumour OR malign*) AND orthodontic*)	**63**
**Web of Science™ Core Collection**	(chemotherapy OR chemo OR ctx OR radiotherapy OR radiation OR irradiation OR “x-ray therapy” OR actinotherapy) AND (cancer OR oncol* OR surviv* OR leukemia OR neoplasm* OR tumor OR tumour OR malign*) AND orthodontic* (Topic) Timespan: All years. Search language=Auto	**181**
**ClinicalTrials.gov**	Condition or disease: Cancer survivor; Intervention/Treatment: Orthodontic treatment	**0**
**ProQuest Dissertations and Theses Global**	title((chemotherapy OR chemo OR ctx OR radiotherapy OR radiation OR irradiation OR “x-ray therapy” OR actinotherapy) AND (cancer OR oncol* OR surviv* OR leukemia OR neoplasm* OR tumor OR tumour OR malign*) AND orthodontic*) OR abstract((chemotherapy OR chemo OR ctx OR radiotherapy OR radiation OR irradiation OR “x-ray therapy” OR actinotheraphy) AND (cancer OR oncol* OR surviv* OR leukemia OR neoplasm* OR tumor OR tumour OR malign*) AND orthodontic*) Filters activated: Full text	**0**

**Table 3:** Excluded records with reasons for exclusion.

Record	Reason for Exclusion
Dahllöf G, Jönsson A, Ulmner M, Huggare J. Orthodontic treatment in long-term survivors after pediatric bone marrow transplantation. Am J Orthod Dentofacial Orthop. 2001 Nov;120(5):459–65.	Case series with fewer than 10 participants
Feghali S, Vi-Fane B, Picard A, Kadlub N. Dental and orthodontic follow-up in nevoid basal cell carcinoma syndrome patient with odontogenic keratocystic tumors. J Stomatol Oral Maxillofac Surg. 2022 Jun;123(3):e57–e61.	No quantitative data
Iatrou I, Vardas E, Theologie-Lygidakis N, Leventis M. A retrospective analysis of the characteristics, treatment and follow-up of 26 odontomas in Greek children. J Oral Sci. 2010 Sep;52(3):439–47.	Benign tumor
Li H, Hu J, Luo E, Zhu S, Li J. Treatment of osteochondroma in the mandibular condyle and secondary dentofacial deformities using surgery combined with orthodontics in adults. J Oral Maxillofac Surg. 2014 Nov;72(11):2295–317.	Benign tumor

**Table 4:** Characteristics of the included studies.

Study	Cancer Survivors’ Characteristics	Orthodontic Tx Characteristics
**Mitus-Kenig et al.** [33] 2015	**40 (17F, 23M); age: nm** **Cancer type:** leukemia [23]; brain tumors [10]; rhabdomyosarcoma [3]; neuroblastoma [2]; lymphoma [2] **Cancer Tx:** chemotherapy, radiotherapy, surgery	**Orthodontic diagnosis:** Skeletal Class I [9]; Class II [26]; Class III [5]; Crowding [31]; High angle cases [8] **Timing from cancer Tx:** nm; **Tx mechanics:** FA; **Extractions:** 2 **Tx duration (average):** 12.5 m **Early conclusion of Tx due to cancer relapse:** 9 **Compromised results:** 6 **Tx complications:** inflammation of the oral mucosa [11]; root resorption [0]
**Mitus-Kenig et al.** [34] 2020	**40 (26F, 14M); age: median 19.4 y (range: 14–28)** **Cancer type:** leukemia [22]; neuroblastoma [3]; soft tissue sarcoma [3]; non-Hodgkin lymphoma [6]; Wilms’ tumor [6] **Cancer Tx:** chemotherapy, radiotherapy	**Orthodontic diagnosis:** Skeletal Class I [9]; Class II [26]; Class III [5] **Timing from cancer Tx:** median 9 y (range: 6–12); **Tx mechanics:** labial FA; MBT; .022” slot; **Extractions:** 4 ***Comparison to a CG matched for age (±4 y), gender and malocclusion*** **Tx duration (average):** 12.5 m [CG: 18 m, *p* < 0.01] **Final w-PAR:** 4.2 [CG: 6.0, *p* > 0.50] **Reduction in w-PAR:** 81.7% [CG: 80.5%, *p* > 0.50] **Tx complications:** inflammation of the oral mucosa: 11 [CG: 5, *p* < 0.05]; root resorption: 3 [CG: 0, *p* < 0.05] **OHIP-14 total score [before Tx, 2w in Tx, 3m in Tx, after Tx]:** *p* > 0.50 **OHIP-14 impacts [before Tx, 2w in Tx, 3m in Tx, after Tx]:** *p* > 0.50
**Mitus-Kenig et al.** [35] 2020	**76 (48F, 28M); age: 19.4 y (13–28)** **Rapid Tx group [RG]:** 36 (26F, 10M); age: 19.4 y (13–28) **Standard Tx group [SG]:** 40 (22F, 18M); age: 19.2 y (14–28) **Cancer type:** leukemia [47]; neuroblastoma [7]; soft tissue sarcoma [5]; non-Hodgkin lymphoma [8]; Wilms’ tumor [9] **Cancer Tx:** chemotherapy, radiotherapy	**Orthodontic diagnosis: [RG]:** Skeletal Class I [10]; Class II [20]; Class III [6]; **[SG]:** Class I [14]; Class II [22]; Class III [4] **Timing from cancer Tx:** [**RG]:** mean 7.8 y (SD: 3.8); **[SG]:** mean 8.6 y (SD: 3.6); **Tx mechanics:** ceramic labial FA; MBT; .022” slot ***Comparisons between RG and SG: Overall final results presented no differences* ^§^** **Tx duration (average):** 11.3 m [SG: 19.3, *p* < 0.01] **Compromised results:** 4 [SG: 2] **Tx complications:** oral mucositis: 6 [SG: 7]; root resorption: 0 [SG: 4] **OHIP-14 total score [before Tx, 2w in Tx, 3m in Tx, after Tx]:** *p* > 0.50 **OHIP-14 impacts [before Tx, 2w in Tx, 3m in Tx, after Tx]:** *p* > 0.50
**Mitus-Kenig et al.** [36] 2021	**52 (23F, 29M); age: 19.4 y (13–28)** **Cancer type:** leukemia [28]; neuroblastoma [4]; soft tissue sarcoma [4]; non-Hodgkin lymphoma [8]; Wilms’ tumor [8] **Cancer Tx:** chemotherapy, radiotherapy	**Orthodontic diagnosis:** Skeletal Class I [13]; Class II [33]; Class III [6] **Timing from cancer Tx:** mean 8.2 y (SD: 4.1); **Tx mechanics:** labial FA; MBT; .022” slot; **Extractions:** 2 [CG: 4]; **Hyrax:** 4 [CG: 5] **Retention protocol:** Fixed retainer 13–23 and 33–43—combined with upper Hawley (night-time only for 1.5 y) ^§§^ ***Comparison to a CG matched for age (±4 y), gender, malocclusion, and Tx duration*** **Tx duration (average):** 17.6 m [CG: 18.1 m, *p* > 0.50] **Before Tx: w-PAR:** 23.0 ± 7.5 [CG: 22.6 ± 6.2, *p* > 0.50]; **ICON:** 62.4 ± 11.9 [CG: 64.0 ± 12.1, *p* > 0.50] **After Tx: w-PAR:** 4.6 ± 2.1 [CG: 4.2 ± 1.9, *p* > 0.50]; **ICON:** 10.2 ± 3.3 [CG: 9.3 ± 4.1, *p* > 0.50]; **PSS**: *p* > 0.50 **At 3-year follow-up: w-PAR:** 6.4 ± 2.4 [CG: 4.9 ± 2.1, *p* = 0.007]; **ICON:** 15.6 ± 2.6 [CG: 10.2 ± 3.2, *p* = 0.004]; **PSS**: *p* > 0.50

CG: control group; F: female; FA: fixed appliances; ICON: index of complexity, outcome and need; M: male; m: months; nm: not mentioned; OHIP-14: oral health impact profile-14; PSS: patient satisfaction score; RG: Rapid Tx duration group—patients whose oncological profile could influence the duration of orthodontic Tx and were expected to complete Tx within 12 m; SG: Standard Tx duration group—patients whose oncological profile did not influence the duration of orthodontic Tx; Tx: treatment; w: weeks; w-PAR: weighted peer assessment rating index; y: years. ^§^ As per the authors assessed on the basis of plaster casts, cephalometric images, and photographic documentation. No other information was provided. ^§§^ Compliance was assessed by questioning parents and patients and checking the appliances’ fit. Patient compliance was similar in both groups. Check-ups within the retention period were performed every 3 months for 1.5 years after the appliances had been removed.

**Table 5:** Risk of bias assessment with the Newcastle-Ottawa Scale.

Criteria	Mitus-Kenig et al. [33] 2015	Mitus-Kenig et al. [34] 2020	Mitus-Kenig et al. [35] 2020	Mitus-Kenig et al. [36] 2021
**Selection**				
Representativeness of the exposed cohort	⭐️	⭐️	⭐️	⭐️
Selection of the non-exposed cohort		⭐️		⭐️
Ascertainment of exposure	⭐️	⭐️	⭐️	⭐️
Demonstration that outcome of interest was not present at start of study	⭐️	⭐️	⭐️	⭐️
**Comparability**				
Comparability of cohort on basis on design or analysis		⭐️⭐️		⭐️⭐️
**Outcome**				
Assessment of outcome	⭐️	⭐️	⭐️	⭐️
Follow-up	⭐️	⭐️	⭐️	⭐️
Adequacy of follow-up	⭐️	⭐️	⭐️	⭐️

## Data Availability

The data underlying this article come from those included in the relevant published articles. The latter will be shared upon reasonable request to the corresponding author.

## Registration

Open Science Framework (https://osf.io/z7mg5/, accessed on 24 March 2024).
